# Auditory Delusional Misidentification: A Case of Capgras Syndrome During the COVID-19 Pandemic

**DOI:** 10.1192/bjo.2024.658

**Published:** 2024-08-01

**Authors:** Michael Shaw, Alana Durrant, Leia Penfold, Opeyemi Okediran, Hideaki Tokuma

**Affiliations:** 1 Norfolk & Suffolk NHS Foundation Trust, Bury St Edmunds, United Kingdom; 2 West Suffolk NHS Foundation Trust, Bury St Edmunds, United Kingdom

## Abstract

**Aims:**

Capgras syndrome is the most common of the delusional misidentification syndromes. It is characterised by the delusional belief that a familiar person has been replaced by an identical imposter. Capgras syndrome is associated with functional conditions, like psychosis, but also with a range of organic conditions such as dementia, brain injury and Parkinson's disease.

The COVID-19 pandemic created a unique situation where patients were unable to see their relatives in person, resulting in their only form of contact being via telephone or virtually.

**Methods:**

We present a case of a 70 year old lady Mrs W, who was admitted with first episode psychosis. She developed Capgras syndrome during her admission, based purely in the auditory modality of talking to her husband on the telephone. She was firm in her conviction that he was an imposter, mainly based of ‘a different tone’ and using ‘different words’. Showing a photograph of her husband was met with full and appropriate recognition. Given that a significant minority of elderly patients with a Capgras delusion have an organic aetiology, neuroimaging and extended laboratory investigations, including auto-antibodies for limbic encephalitis, were performed which were unremarkable.

With psychotropic medication, the Capgras delusion resolved, and on discharge she recognised her husband when they met again.

**Results:**

The aetiology of Capgras syndrome remains unclear, although a range of causes have been suggested. Early psychodynamic theories related to conflict between love and hate towards the relative, which could be relevant in functional conditions but may have less significance in organic conditions.

Other theories examine Capgras syndrome as a mirror of prosopagnosia, where people have difficulties recognising familiar faces. This would indicate a pathological process affecting visual pathways. However, our case challenges this theory, suggesting that deficits in other sensory modality pathways may also contribute.

Although rare, our case is not entirely unique; several cases of Capgras syndrome in people with blindness have been reported. However, our case differs in that our patient was able to recognise photos of her husband despite misidentification based on auditory cues. As Mrs W did not have visual impairment, it is unclear if she would have presented with the more classical visual misidentification in the absence of the unique circumstances of the COVID-19 pandemic.

**Conclusion:**

Capgras syndrome is classically associated with misidentification based on visual cues, however a growing number of case reports challenge this. Further investigation is required to create theories that encompass other sensory modalities.

